# Comparison of Post-auricular Soft Tissue and Post-auricular Soft Tissue With Autologous Bone Pate in Obliteration of the Canal Wall Down Mastoidectomy Cavity

**DOI:** 10.7759/cureus.75734

**Published:** 2024-12-15

**Authors:** Bosco Suriya Luke Rathnakumar, K. C Prasad, Charuvi Guttal, Gautham S.

**Affiliations:** 1 Department of Otolaryngology, Head and Neck Surgery, Sri Devaraj Urs Academy of Higher Education and Research, Kolar, IND

**Keywords:** canal wall down mastoidectomy, cavity problems, cholesteatoma, chronic otitis media, tympanoplasty

## Abstract

Background

The surgical management of chronic otitis media (COM) with squamous disease is canal wall down mastoidectomy (CWDM). Canal wall down procedures require the obliteration of the newly formed cavity to mitigate complications. Soft tissue flaps, including Rambo flap, Hong Kong flap, Palva flap, and inferior-based fascio-periosteal flap, as well as autologous bone pâté, have been the most successful and commonly used materials for obliteration over the past two decades. Although each flap has its advantages, the Palva flap is considered superior, primarily because of its ease of use and the simplicity of its design.

In this study, we intend to evaluate the effectiveness of mastoid obliteration using autologous bone pate with Palva flap compared to Palva flap (post-auricular fibro-periosteal soft tissue) in patients who underwent CWDM.

Methods

Sixty-two patients with COM with squamous disease were included in the study. The patients underwent CWDM with tympanoplasty and meatoplasty, followed by cavity obliteration using two different methods: group A - obliteration with Palva flap (post-auricular fibro-periosteal soft tissue) and group B - obliteration with Palva flap and bone pate. The patients were evaluated for cavity problems on post-operative days 21, 30, 60, 90, and 180, based on a 10-point scale compiled from the literature.

Results

Our study found that the majority of patients had right-sided disease and were between 41 and 60 years of age. Five out of sixty-two (8.1%) patients developed cavity problems, three in group A (9.7%) and two in group B (6.5%). The incidence of cavity problems was almost the same in both groups. The cavity problems were as follows: vertigo (1/62, 1.6%), surgical site infection (2/62, 3.2%), and graft failure (2/62, 3.2%). However, there were no statistically significant differences (p-value: 0.62) between either of the obliteration methods in terms of mitigating cavity problems.

Conclusion

Post-auricular soft tissue and post-auricular soft tissue with autologous bone pate in the obliteration of the CWDM cavity provided similar results in terms of mitigating cavity problems.

## Introduction

Chronic otitis media (COM) is one of the most commonly encountered middle ear pathologies. COM is defined as an inflammatory process in the middle ear space that results in long-term or permanent changes in the tympanic membrane, including atelectasis, dimeric membrane formation, tympanosclerosis, formation of a retraction pocket, or cholesteatoma with varying degrees of damage to the ossicular chain [1]. The worldwide prevalence of COM is about 65 to 330 million, with 60% of patients experiencing significant hearing loss [2-4]. In India, the prevalence of chronic suppurative otitis media in children has been reported to be 3.78% and around 7.7% among others [3]. This presents significant morbidity for patients, as the symptoms usually include a discharging ear, which may be foul-smelling, aural fullness, otalgia, hearing loss causing social embarrassment, and tinnitus, all of which may hinder the patient's social and professional life. Otitis media is classified into acute or chronic based on the disease's duration, inactive and active disease based on the presence or absence of discharge, and mucosal and squamous disease based on the presence or absence of cholesteatoma [4-8]. COM is not the result of the non-resolution of acute suppurative otitis media (ASOM), as previously thought, although an acute infection may aggravate it [5]. The reason certain patients progress from an acute to chronic infection and why others experience spontaneous resolution remains unclear, with various genetic and non-genetic factors currently under research [6].

Based on our clinical experience, the incidence of COM with squamous disease in the Kolar district of Karnataka is generally high, with lower socioeconomic status and poor oral hygiene likely being the most predominant etiologies. This, therefore, necessitates a canal wall down procedure for the surgical management of the disease. However, a canal wall down mastoidectomy (CWDM) is associated with cavity problems, which pose significant morbidity to the patient. For instance, frequent follow-up visits for debris clearance, regular monitoring of the cavity for recidivism or recurrence, higher susceptibility to ear infections, and vertigo upon exposure to extremes of temperature. It has been well-established in the literature that the incidence of cavity problems can be reduced or prevented by obliterating the mastoidectomy cavity. Various materials have been used in the literature, such as bone pate, bioactive glass, post-auricular fibro-periosteal soft tissue, hydroxyapatite, titanium, and silicone [9].

Aim 

In this study, we aimed to compare the cavity problems encountered using two different materials: group A - post-auricular fibro-periosteal soft tissue, and group B - autologous bone pate with post-auricular fibro-periosteal soft tissue for mastoid cavity obliteration in patients undergoing CWDM. We also aimed to compare the efficacy of these two methods in reducing cavity problems.

Objectives

The objectives of this study are: 1) to evaluate the outcomes of mastoidectomy cavity obliteration using post-auricular fibro-periosteal soft tissue alone following CWDM in group A patients with squamosal type COM; 2) to assess the outcomes of mastoidectomy cavity obliteration using autologous bone pâté with post-auricular fibro-periosteal soft tissue following CWDM in group B patients with squamosal type COM; and 3) to compare post-operative outcomes in terms of cavity problems between groups A and B.

## Materials and methods

The sample size for this prospective interventional study was estimated to be 62 patients with COM-squamous disease, considering an alpha error of 5% and 80% power, based on the prevalence of COM (4%) from a study by Ghiasi et al. [10]. The study was conducted from July 2022 to January 2023 and included patients of either gender, aged 18-65 years, with proven COM who underwent canal wall down mastoidectomy with obliteration of the mastoidectomy cavity using either post-auricular soft tissue or post-auricular soft tissue with bone pâté. Exclusion criteria included patients with very extensive disease leading to the formation of post-auricular fistulas, extensive disease where there was a likelihood of obliterating diseased cells, patients undergoing revision ear surgery, immunosuppressive therapy, or those with a history of chemotherapy or radiation therapy. Additionally, patients with aural polyps, pre-existing vertigo, Meniere's disease, benign paroxysmal positional vertigo (BPPV), or perilymph fistulas were excluded. The follow-up period for the study was six months.

Patients with COM with squamous disease who met the inclusion criteria were enrolled in this study after providing informed written consent. They were treated according to the current standard of care: canal wall down mastoidectomy with obliteration, performed either using post-auricular fibro-periosteal soft tissue alone or in combination with bone pate. Routine otoscopy and radiological examinations, including high-resolution computed tomography (HRCT) of the temporal bone, were conducted. The CT scan was used to plan the surgical dissection limits, assess the continuity of the ossicular chain, evaluate mastoid pneumatization, identify facial nerve dehiscence, determine the extent of the cholesteatoma matrix, and note anatomical variations, such as a high jugular bulb or an anteriorly placed sigmoid sinus. Based on these findings, patients were stratified into two obliteration arms. If the patient had a sufficient amount of cortical bone or the disease had not eroded the cortical bone of the mastoid, they were assigned to group B; otherwise, they were assigned to group A. The study population, consisting of 62 patients, was thus evenly divided into two treatment arms of 31 patients each.

**Figure 1:**
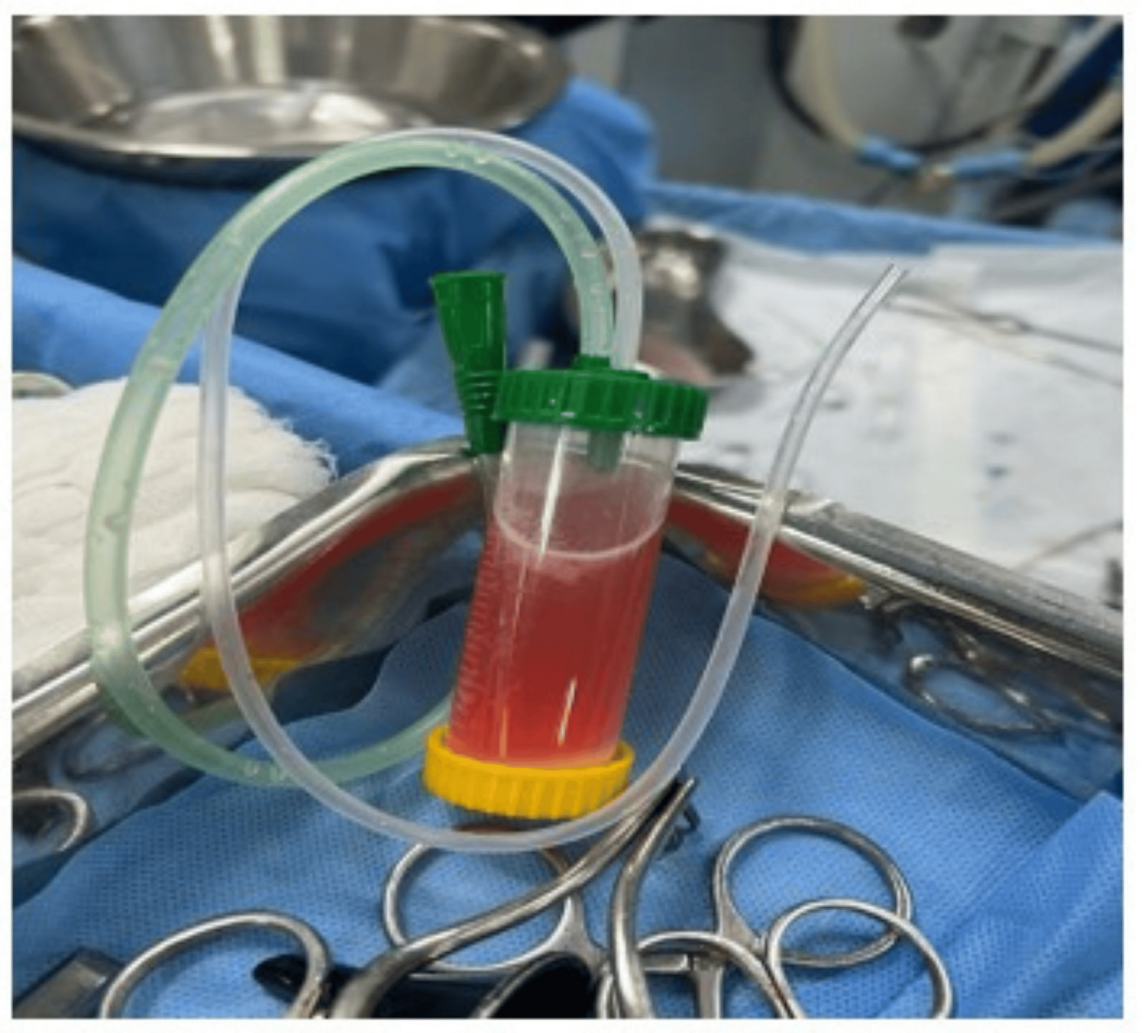
Romsons Mucus Extractor®

The patients underwent conventional canal wall down mastoidectomy, type III tympanoplasty by underlay technique and single-stage ossicular reconstruction by homologous septal spur cartilage, obliteration of the mastoidectomy cavity, and meatoplasty in that order. A clear informed consent was taken for the use of homologous tissue for ossicular reconstruction. We preferred a temporalis fascia graft for tympanoplasty. When obliteration with bone pate was planned, a mucus extractor (Figure 1) was used to suction the mastoid and collect bone dust while drilling. The suctioned material contained a mixture of saline and bone dust, of which the latter precipitated to the bottom. The supernatant saline was discarded and the bone dust was washed with fresh, sterile 09% normal saline, mixed with gentamicin (80 mg in 2 mL, Genticyn®), and set aside till the obliteration stage of the procedure. If not, the post-auricular fibro-periosteal soft tissue was harvested, pressed, and flattened using House's graft press forceps. The areas requiring obliteration included the sino-dural angle, supra-labyrinthine area, mastoid tip, and retro-facial region. The areas to be obliterated were identified, cleared of disease, and obliterated with post-auricular soft tissue or post-auricular soft tissue combined with bone pate, based on the extent of the disease and mastoid pneumatization as determined by the CT scan. The external auditory canal was packed with gel foam soaked in Polymyxin B (10,000 IU/g) and Neomycin (3,400 IU/g) (Neosporin-HTM) drops, followed by a sterile cotton pack soaked in Polymyxin B (5,000 units), Neomycin (3,400 units), Bacitracin (400 units), and Hydrocortisone (10 mg) (Neosporin-HTM) ointment. The surgical wound was closed in layers using 3-0 polyglactin 910 (Vicryl-RB) for the subcutaneous layer and 3-0 nylon (Ethilon-RC) with simple interrupted sutures for the cutaneous layer. A sterile mastoid dressing was applied over the operated ear.

Following surgery, the patients were administered a course of intravenous antibiotics (Amoxicillin + Clavulanic acid, 1.2 grams twice daily) for five days, in accordance with the institution's antibiotic policy, and were typically discharged on the seventh post-operative day with oral antibiotics and antihistamines to complete a 14-day course. The patients were scheduled for follow-up visits on the 21st, 30th, 60th, and 90th post-operative days, with a total follow-up period of six months. On the 14th post-operative day, the post-auricular area was inspected for any gaping or surgical site infection, and the sutures were removed. During the 21st post-operative day (third-week) visit, the canal pack was removed, and any excess gel foam was suctioned out. The cavity was examined for discharge, granulation, debris, slough, or remaining gel foam, which was suctioned as needed. Patients were subsequently re-examined on the 30th, 60th, and 90th post-operative days and evaluated using a 10-point scale (Table 1).

**Table 1:** Evaluation parameters for patients undergoing canal wall down mastoidectomy

Sr. no	Parameter	(+1)	(0)
1	Epithelialization of the mastoidectomy cavity	Good	Poor
2	Meatoplasty	Normal	Stenosed
3	Post-op wound infections	Absent	Present
4	Discharge	Absent	Present
5	Vertigo	Absent	Present
6	Perichondritis	Absent	Present
7	Uniformity of the mastoidectomy cavity	uniform	Non-uniform with steep edges/ledges
8	Granulation tissue in the mastoidectomy cavity	Absent	Present
9	Visualization of the whole mastoidectomy cavity by gentle retraction of the pinna	Visualized	Not visualized
10	Graft uptake	Good	Infection/failure
Total score:			

The patients were awarded one point for each favorable outcome and none for an unfavorable outcome. For example, if a patient experienced post-operative vertigo but all other parameters were normal, the final score would be 9/10. If the patient experienced both vertigo and graft failure post-operatively, the final score would be 8/10, and so on.

Statistical analysis

Data were entered in Microsoft Excel and analyzed using SPSS 22.0 version software (IBM, Armonk, NY). Categorical data were represented as "Frequencies" and "Proportions." A paired t-test and the Kruskal-Wallis test were used to assess significant differences between the means of two paired measurements: post-auricular soft tissue alone and post-auricular soft tissue combined with bone pate.

## Results

**Figure 2:**
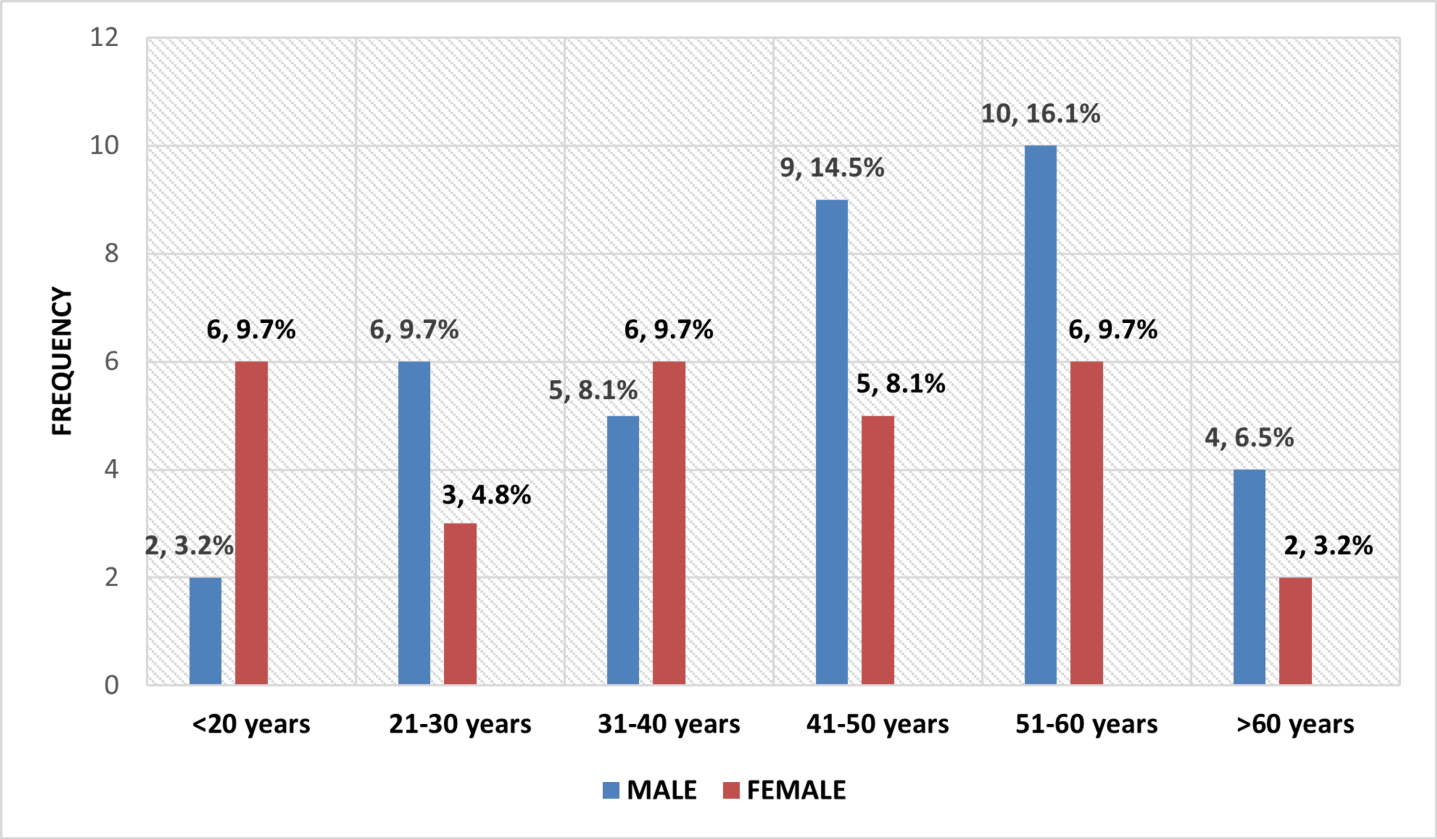
Distribution of subjects according to age group and gender

The study population included a mixture of males and females. The majority of the patients in our study (30/62, 48.4%) were between 41 and 60 years old. In all age groups, it was observed that the number of males (36/62, 58.1%) exceeded the number of females (26/62, 41.9%) affected by the disease as seen in Figure 2.

Out of the 62 patients studied, the majority had the right-sided disease (39/62; 62.9%) compared to the left (21/62; 33.9%), and only a small number (2/62, 3.2%) had bilateral disease, as seen in Table 2. None of the patients had any pre-existing intracranial or extracranial complications and none presented with facial nerve weakness.

**Table 2:** Distribution of subjects according to site COM, chronic otitis media

Location of the COM-squamous disease	Frequency	Percentage
Right	39	62.90
Left	21	33.87
Bilateral	02	3.23
Total	62	100.00

Based on the EAONO/JOS classification of cholesteatoma (refer to Table 7 in Appendices) [11], the patients were categorized into four grades of cholesteatoma, as detailed in Table 3: Grade 1 - cholesteatoma limited to the primary site; Grade 2 - cholesteatoma involving two or more sites; Grade 3 - cholesteatoma with extracranial complications or adhesive otitis media; and Grade 4 - cholesteatoma with intracranial complications. This classification primarily aided in determining the urgency of surgery and the extent of surgical exploration.

**Table 3:** Distribution of subjects according to disease staging

Staging of the disease	Frequency	Percentage
Grade I	32	51.61
Grade II	24	38.71
Grade III	06	9.68
Grade IV	00	0

The areas targeted for obliteration of the mastoid cavity included the sino-dural area, supra-labyrinthine area, mastoid tip, and retro-facial area. The specific areas obliterated varied significantly due to the diverse pneumatization patterns of the mastoid. The frequency of patients undergoing obliteration of these areas is presented in Table 4. However, the number of areas obliterated had no impact on mastoid cavity epithelialization, graft uptake, or cavity-related issues.

**Table 4:** Distribution of subjects according to number of areas obliterated

Areas obliterated	Frequency	Percentage
One area only	00	0
Two areas	25	40.32
Three areas	33	53.23
Four areas	04	6.45

On post-operative days 30, 60, 90, and 180, the mastoid cavity was evaluated using the 10-point assessment scale. We observed that 5/62 patients (8.06%) developed cavity-related problems, including graft failure (2/62, 3.2%), post-operative wound infections (2/62, 3.2%), and vertigo (1/62, 1.6%). The frequency of these issues in each group is presented in Table 5.

**Table 5:** Cavity problems observed in groups A and B

S. No.	Evaluation of the mastoid cavity	Frequency of cavity problems in group A	Percentage	Frequency of cavity problems in group B	Percentage
1	Poor epithelialization of the mastoidectomy cavity	0	0	0	0
2	Stenosis of the meatoplasty	0	0	0	0
3	Post-operative wound infections	1	1.61%	1	1.61%
4	Discharge	0	0	0	0
5	Perichondritis	0	0	0	0
6	Vertigo	1	1.61%	0	0
7	Uniformity of the mastoidectomy cavity	0	0	0	0
8	Granulation tissue in the mastoidectomy cavity	0	0	0	0
9	Poor visualization of the whole mastoidectomy cavity by gentle retraction of the pinna	0	0	0	0
10	Poor graft uptake	1	1.61%	1	1.61%

The aggregate mean score of patients in Group A was 9.903 with a standard deviation of 0.301, while the mean score in Group B was 9.935 with a standard deviation of 0.250. The scores for each group were analyzed using the paired t-test and Kruskal-Wallis test. The aggregate H-value was calculated to be 0.25, with a p-value of 0.62 (Table 6), indicating that the statistical difference between the two groups was insignificant. Thus, there is no significant difference between the two obliteration methods. Accordingly, the null hypothesis, which states that there is no statistically significant difference between the outcomes of mastoidectomy performed with obliteration using post-auricular fibro-periosteal soft tissue alone versus post-auricular fibro-periosteal soft tissue combined with bone pate in canal wall down mastoidectomies, can be accepted.

**Table 6:** The aggregate scores of patients in groups A and B

S. No.	Evaluation parameter	Group A	Group B	H-value	p-value
1	Poor epithelialization of the mastoidectomy cavity	0/31	30/31	0	0.99
2	Stenosis of the meatoplasty	0/31	0/31	0	0.99
3	Post-operative wound infections	1/31	1/31	0	0.99
4	Discharge	0/31	0/31	0	0.99
5	Perichondritis	0/31	0/31	0	0.99
6	Vertigo	1/31	0/31	0.04	0.85
7	Uniformity of the mastoidectomy cavity	0/31	0/31	0	0.99
8	Granulation tissue in the mastoidectomy cavity	0/31	0/31	0	0.99
8	Poor visualization of the whole mastoidectomy cavity by gentle retraction of the pinna	0/31	0/31	0	0.99
10	Poor graft uptake	1/31	1/31	0	0.99
H-value: 0.25; p-value: 0.62					

## Discussion

COM is a disease with multifactorial etiopathogenesis, including toll-like receptors, cytokine-mediated innate immune responses, immunoglobulin-mediated inflammation, and craniofacial maldevelopments [10,11]. It is generally accepted that COM with squamous disease is localized to the squamous epithelium in the epitympanum and the posterior mesotympanum [12]. In developing countries, poverty, congested households, malnutrition, exposure to smoke, and lack of hygiene have been identified as prime causes of COM [13-15]. This is further substantiated by a 10-year nationwide study done with Māori children, which found that COM’s incidence dropped by 50% when there were significant improvements in the housing conditions, nutritional status, and hygiene of the local populace [16]. Surveys conducted in Southeast Asian developing countries showed that COM is the most common cause of moderate hearing loss (40-60 dB hl) [17]. In the year 2002 alone, it was associated with a morbidity of 1.5 disability-adjusted life years (DALY) [18]. All of these studies indicate that COM is a disease of significant burden on the population and the healthcare setups in developing countries.

The surgical treatment of COM with squamous disease is a CWDM. However, CWDM is associated with significant cavity problems such as vertigo, discharge, and granulation tissue. The reason for these problems is that the now wide-open mastoid cavity, if left uncovered, exposes the semicircular canals to the external environment, causing vertigo, and allows the mastoid bone to secrete exudative fluid [19]. There is no shortage of literature to suggest that the obliteration of the mastoidectomy cavity is an effective, suitable, and convenient method to circumvent cavity problems [20-24]. Another significant problem encountered in developing countries is the weaker economic status of the general populace, unsupportive infrastructure, and poor post-operative compliance, which limit the surgeon's ability to use more expensive materials for obliteration, such as S53P4 bioactive glass or titanium chips. This also forces the surgeon to work with cheaper, well-established materials to achieve favorable outcomes. The need for a self-cleaning and well-epithelialized cavity becomes even more critical due to poor patient compliance, which is why autologous materials were chosen in our study. These materials were readily available, easily moldable, and carried no risk of adverse reactions. However, there was always the risk of including diseased bone in the bone pate. Hence, patient selection was done meticulously; if any bony erosion was suspected, the patient was included in the post-auricular soft tissue obliteration group. 

Our study used postauricular fibro-periosteal soft tissue in group A and Palva flap with bone pate (harvested while drilling the cortical bone) for mastoid cavity obliteration in group B. The incidence of cavity problems was almost the same in both cases (3/62, 4.83% in group A and 2/62, 3.22% in group B). In the single patient who developed vertigo, the cholesteatoma covered the endosteal layer of the oval window. The vestibule was exposed upon removal of the cholesteatoma and, therefore, had to be plugged by soft tissue, causing post-operative vertigo. The two patients who developed surgical site infections had been diagnosed with type II diabetes mellitus post-admission, which probably contributed to poorer, inadequate healing outcomes. This also made those patients more susceptible to acquired infections than the general populace.

All the patients were followed up regularly for at least six months and longer in graft failure cases where a wait-and-watch policy was adopted wherein the graft eventually took up after one year. We noted that irrespective of the obliteration method, the epithelialization, uniformity, and visualization of the mastoidectomy cavity were almost equivalent in both groups. 

Limitations

The limitations of the study were its relatively smaller population size and a short follow-up period. This study did not account for the presence of comorbidities, which may hinder adequate wound healing and result in a higher rate of cavity problems. Additionally, the study lacked a comparison with other obliteration methods, such as synthetic materials, which could have provided valuable insights into the most effective techniques for reducing cavity problems.

## Conclusions

The rates of post-mastoidectomy cavity problems were similar between the two groups. Both groups showed comparable outcomes in terms of epithelialization, cavity uniformity, and the presence of granulation tissue. These findings align with those reported in other published studies. Therefore, it can be safely concluded that mastoid obliteration using autologous bone, in addition to post-auricular soft tissue, is a safe and effective material for obliteration in canal wall down procedures.

## Appendices

**Table 7:** EAONO/JOS joint consensus on the classification and staging of cholesteatoma Yung M, Tono T, Olszewska E, et al.: EAONO/JOS joint consensus statements on the definitions, classification and staging of middle ear cholesteatoma. J Int Adv Otol. 2017 Apr 1;13(1):1-8. DOI: 10.5152/iao.2017.3363 (Reproduced with permission from Matthew Yung) [11].

Sr. no	Stage	Description
1	Stage I	Cholesteatoma localized in the primary site: attic/tympanic cavity. The site of cholesteatoma origin is to be indicated by the following abbreviations: the attic (A) for pars flaccida cholesteatoma. The tympanic cavity (T) for pars tensa cholesteatoma, congenital cholesteatoma, and cholesteatoma secondary to a tensa perforation.
2	Stage II	Cholesteatoma involving two or more sites.
3	Stage III	Cholesteatoma with extracranial complications or pathologic conditions including facial palsy, labyrinthine fistula, labyrinthitis, post-auricular abscess or fistula, zygomatic abscess, neck abscess, canal wall destruction involving more than half the length of the bony ear canal, destruction of the tegmen with a defect that requires surgical repair, and adhesive otitis media causing total adhesion of the pars tensa.
4	Stage IV	Cholesteatoma with intracranial complications including purulent meningitis, epidural abscess, subdural abscess, brain abscess, sinus thrombosis, and brain herniation into the mastoid cavity.
